# Genetic Diversity and Population Structure of Endangered Orchid *Cypripedium flavum* in Fragmented Habitat Using Fluorescent AFLP Markers

**DOI:** 10.3390/plants13202851

**Published:** 2024-10-11

**Authors:** Shijun Hu, Meizhen Wang, Xiaohui Yan, Xiaomao Cheng

**Affiliations:** 1 Key Laboratory of National Forestry and Grassland Administration on Biodiversity Conservation in Southwest China, Southwest Forestry University, Kunming 650224, China; shijunhu@swfu.edu.cn (S.H.); wmz20241003@126.com (M.W.); 2 Key Laboratory of Forest Disaster Warning and Control of Yunnan Province, Southwest Forestry University, Kunming 650224, China; 3 Southwest Landscape Architecture Engineering Research Center of State Forestry and Grassland Administration, College of Landscape Architecture and Horticulture Sciences, Southwest Forestry University, Kunming 650224, China

**Keywords:** *Cypripedium flavum*, genetic diversity, population structure, AFLP

## Abstract

Genetic diversity is crucial for determining the evolutionary potential of a species and is essential for developing optimal conservation strategies. The impact of habitat fragmentation on the genetic diversity of food-deceptive orchids seems to be unpredictable because of their specialized seed and pollen dispersal mechanisms. The habitat of deceptive *Cypripedium flavum* was severely fragmented during the past half century. This study investigated the genetic diversity and structure of seven fragmented *Cypripedium flavum* populations in Shangrila County using AFLP markers. A total of 376 alleles were identified, with a range of 70 to 81 alleles per locus. The species exhibited considerable genetic diversity, as evidenced by an average Nei’s gene diversity (*H*) of 0.339 and a Shannon’s information index (*I*) of 0.505, with all loci being polymorphic. Based on Molecular Variance (AMOVA), 8.75% of the genetic differentiation was found among populations, while the remaining 91.25% of genetic variation occurred within populations. Population structure analysis revealed that the *C. flavum* germplasm can be categorized into 2 distinct groups, among which there was significant gene flow. Despite habitat fragmentation, *C. flavum* still retained a high level of genetic diversity, and the substantial gene flow (5.0826) is a key factor in maintaining the genetic diversity. These findings offer valuable insights for the conservation and potential use of *C. flavum* genetic resources.

## 1. Introduction

The genetic diversity within a species results from long-term evolutionary processes and plays a pivotal role in its survival and adaptive capacity, particularly in response to dynamic climates, environments, and anthropogenic alterations [1]. Comprehending the extent of genetic diversity is crucial to designing effective conservation strategies for the endangered species [2]. Genetic diversity is closely associated with population fitness [3,4], and populations exhibiting low genetic diversity may experience reduced fitness in various environments [5]. While many species exhibit varying levels of genetic diversity to adapt to different environments, habitat fragmentation due to environmental changes or human interference poses significant threats to plant populations, potentially resulting in decline, extinction, and a broader threat to global biodiversity [6,7].

Habitat fragmentation is anticipated to reduce genetic variability and enhance inter-population genetic divergence by increasing genetic drift, inbreeding, and decreasing gene flow [8]. These genetic alterations can decrease individual fitness, impair populations’ ability to adapt to changing selection pressures [9], and decompose large habitats into small, usually isolated patches resulting in the overall reduction in population size and numbers [10]. Genetic diversity can indicate the ecological interactions and processes that have shaped the species’ history [11]. At the population level, genetic studies can be used to identify regions or populations of high conservation priority [6]. However, the effects of habitat fragmentation on population genetics have been studied for several decades, and no clear response patterns have emerged [8]. Species with different dispersal capacities have different responses to habitat fragmentation, for example, orchids tend to exhibit relatively low levels of genetic differentiation, even among widely disjunct populations, because their minute, wind-dispersed seeds are capable of traversing long distances, sometimes over hundreds of kilometers [12].

Gene flow can counteract the loss of genetic diversity caused by small populations in fragmented habitats [13], and molecular markers and genetic diversity analysis provide convenience for studying gene flow. The genetic diversity of some orchids has been analyzed successfully using various molecular markers, such as chloroplast DNA [10,14], nuclear genome ITS sequence [15], amplified fragment length polymorphism (AFLP) [16], inter simple sequence repeat (ISSR) [17], and SSR [18,19,20]. However, AFLP markers offer several advantages over other molecular markers, such as high reproducibility and significant polymorphism, without the need for prior DNA sequence information [21], and high coverage of the genomes in eukaryotic species [22]. Owing to these advantages, AFLP markers are favored in genetic diversity studies [23,24,25].

The lady-slipper orchid, *Cypripedium* species, is a temperate terrestrial orchid and is widely found in Eurasia and North America. Because of their high horticultural value for their beautiful flowers, many *Cypripedium* species are collected illegally from wild populations and, as a result, are threatened with extinction [26]. They are flagship species in conservation biology today. This genus consists of about fifty-nine known species and two varieties [27]. The genus *Cypripedium* is recognized by its slipper-shaped pouch flowers, which are adapted to attract and trap pollinators by deceit [28]. There are technical difficulties associated with their ex-situ cultivation and artificial propagation, which resulted in increased collection pressure and the subsequent rarity of the *Cypripedium* species.

Many orchids face the immediate threat of extinction [11]. *Cypripedium flavum* is endemic to northwestern Yunnan, southeastern Tibet, Sichuan, southern Gansu and western Hubei in high-altitude areas [29]. *C. flavum* is a typical non-rewarding orchid. Owing to the destruction of habitats and anthropogenic activities, the existence of *C. flavum* within its indigenous territory is endangered. Consequently, it has been classified as a nationally protected species [29,30]. Northwest Yunnan is a hotspot of global biodiversity and the origin of many unique taxa. Shangrila is located in the center of this area, with a higher altitude and a fragile ecosystem, and much of Shangrila is not well protected. The habitat of *C. flavum* was fragmented in the 1950s because of massive deforestation in Shangrila, and now, most of the populations are small, which increases inbreeding and the potential for genetic drift. However, for food-deceptive orchid species, habitat fragmentation effects appear especially unpredictable because of their highly specialized seed and pollen dispersal mechanisms [13]. Deceptive pollination may serve as an effective means to maintain high gene diversity within and to counter high genetic differentiation among small orchid populations [31]. In order to protect the deceptive orchids threatened by habitat fragmentation, it is important to know the pattern of the genetic diversity and understand the response to fragmentation. In this study, we study the genetic diversity and population structure among seven fragmented populations of *C. flavum* in Shangrila. Our primary objectives included the following: (1) assessing the genetic diversity and structure both among and within populations, and (2) delineating the degree of genetic differentiation across populations.

## 2. Results

### 2.1. AFLP Polymorphism

A total of 376 distinct alleles were produced by five AFLP primer pairs, all of which (100%) displayed polymorphic patterns. These PCR products were used to analyze a collection of 150 genotypes (Table 1). The number of alleles per primer pair varied between 74 and 81, with an average of 75.2. The five AFLP primer combinations produced 24652 PCR products, with an average of 4925 per primer combination. The average number of PCR products per allele ranged from 60.42 to 70.97, with an average of 66.54 PCR products. The polymorphisms of all primer combinations were 100%, and the discrimination power value ranged from 18.62 to 21.54. The polymorphic information content (PIC) values of five primer combinations ranged from 0.490 to 0.494 with an average of 0.492. As the expected maximum PIC value was 0.5 for a bi-allelic locus, all five primer combinations had PIC values close to 0.5, which indicates that AFLP marker is suitable for studying genetic diversity of *C. flavum* due to high discriminatory power.

### 2.2. Genetic Diversity

Among the seven populations, the number of observed alleles (*N_a_*) ranged from 1.771 to 1.976, averaging 1.914. The number of effective alleles (*N_e_*) ranged from 1.550 to 1.613, averaging 1.583. The Shannon’s information index (*I*) varied between 0.329 and 0.529, with an average of 0.505. The Nei’s gene diversity index (*H*) ranged from 0.324 to 0.355, averaging 0.339. The percentage of polymorphic loci ranged from 88.03% to 98.14%, averaging 94.15%. The Müller index of diversity (*M_u_*) ranged from 0.348 to 0.372, averaging 0.358 (Table 2).

### 2.3. Molecular Variance Analysis

The genetic differentiation among *C. flavum* populations was found to be relatively low, with an *F_st_* value of 0.0896, indicating that only 8.96% of genetic variation exists between populations. Conversely, a high level of genetic variation, accounting for 91.06%, occurs within populations, as suggested by the inbreeding coefficient within populations (*F_is_* = 0.3258). The overall inbreeding coefficient (*F_it_*) for the total population was calculated to be 0.2966. Furthermore, an Analysis of Molecular Variance (AMOVA) indicated that a substantial proportion of the genetic variation, 91.25%, was present within populations, with a minor fraction of 8.75% distributed among populations. The gene flow (*N_m_*), measured at 5.0826, indicated substantial gene migration among the *C. flavum* populations, as summarized in Table 3.

### 2.4. Genetic Structure of C. Flavum

**Figure 1:**
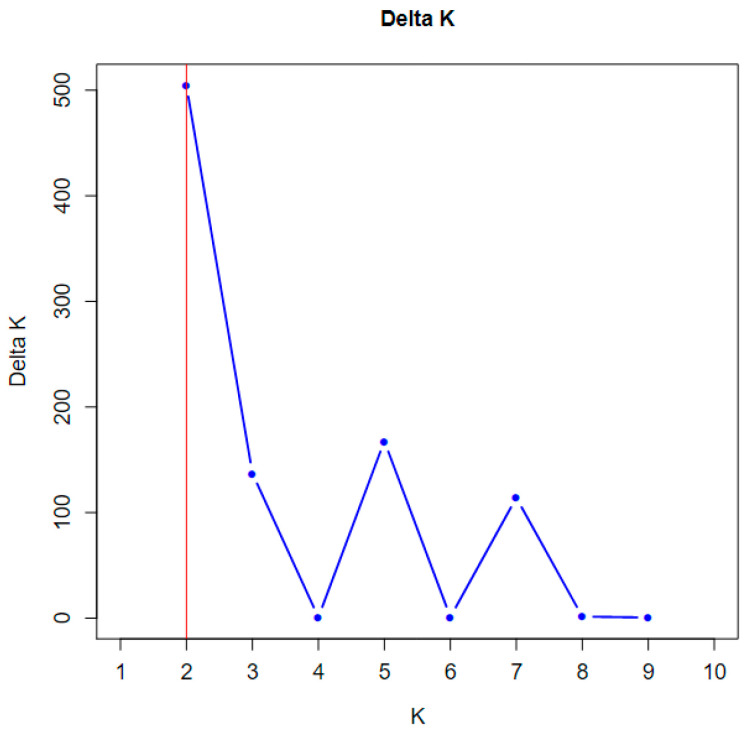
Determinations of genetic groups (K) of *C. flavum* population.

Genetic groups were classified based on the Hierarchical Island model [32]. The model suggests that the K value closest to the peak of ΔK approximates the true number of distinct genetic groups. The largest ΔK was observed when K equaled 2 (Figure 1), leading to the conclusion that there are 2 genetic groups.

**Figure 2:**
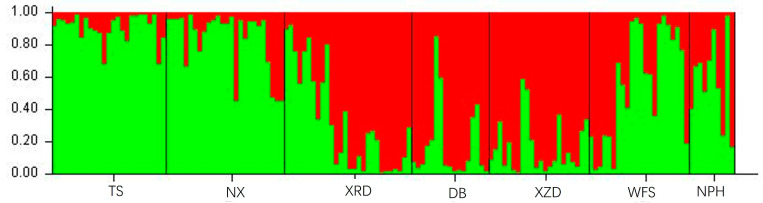
Q value distribution of *C. flavum* K = 2. The 150 individuals were divided into 2 genetic groups. Different color means different genetic groups.

Utilizing the drawing module in STRUCTURE 2.3, a histogram was generated to illustrate the distribution of Q values corresponding to the optimal population structure (Figure 2). The analysis revealed that the minimum Q value occurred at K = 2, suggesting the classification of the 150 individuals into 2 genetic groups. Under K = 2, almost all individuals are genetically mixed, and populations XRD, DB, and XZD were genetically distinguishable from the rest of the populations in the STRUCTURE analysis. The findings suggest that gene flow occurred between the 2 genetic groups.

### 2.5. Genetic Relationship of C. Flavum

**Figure 3:**
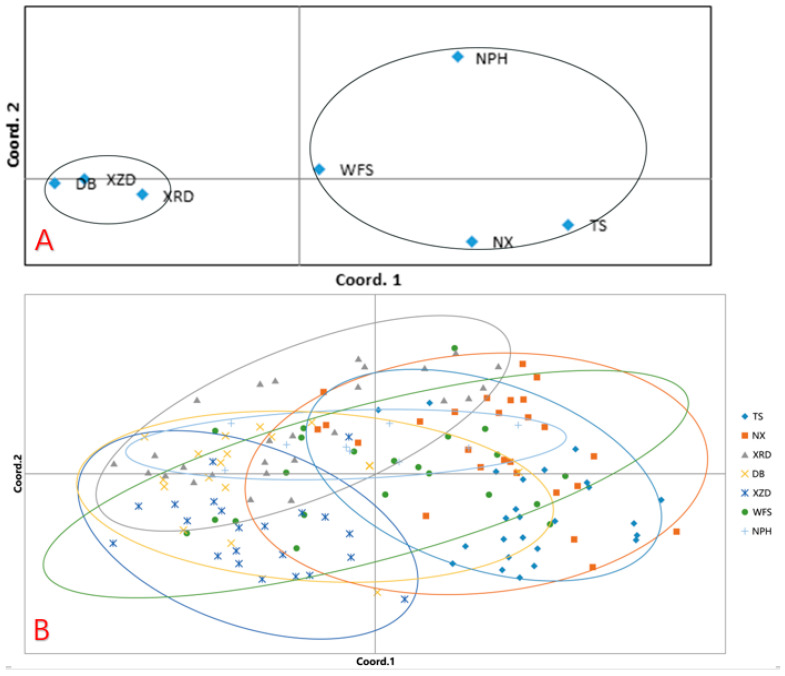
Principal coordinate analysis using AFLP markers and separation on a two-dimensional diagram. (**A**) PCA at the population level; (**B**) PCA at the individual level.

Baseline coordinate analysis based on Nei’s neutral genetic distance was performed. Principal component analysis at population level showed that the first three principal coordinates accounted for 32.86%, 27.23%, and 17.10% of the genetic diversity, cumulatively explaining 77.18% of the total diversity. While the populations were not entirely distinct in the two-dimensional plot derived from the first two principal components, they could be divided into two genetic groups, where one is composed of DB, XZD, and XRD populations, and the other is composed of NPH, WFS, NX, and TS populations (Figure 3A). Principal component analysis at the individual level showed that the sampling individuals were not genetically distinct (Figure 3B).

Among the seven populations of *C. flavum*, the genetic distances between any two given populations ranged from 0.052 to 0.125, with an average of 0.064 (Table 4). The smallest genetic distance was observed between NX and TS (0.052), indicating a minimal degree of genetic differentiation. The greatest was observed between NPH and XZD (0.125), indicating a large degree of genetic differentiation.

## 3. Discussion

### 3.1. Genetic Diversity

Genetic diversity is critical to the long-term survival of species and reflects the potential to adapt to environmental change [33,34]. The genetic diversity within a species is crucial for its ability to adapt to a changing environment [35]. Genetic diversity is quantifiable through several parameters including the effective number of alleles, Nei’s genetic distance, and the Shannon information index, which together illustrate the pattern of genetic variation [36]. In contrast to the polymorphic locus frequencies observed in other species like *C. goeringii* at 84.89% [15], and *C. kanran* at 79.67% [37], the genetic diversity of *C. flavum* is relatively higher (100.00%). Examination of these markers in the *C. flavum* population revealed that the level of genetic diversity (*H_e_* = 0.339) (Table 2), compared with several other species of *Cypripedium*, was significantly lower, such as *Cypripedium calceolus* (*H_e_* = 0.572) [10], *Cypripedium tibeticum* (*H_e_* = 0.745 ± 0.119), and *Cypripedium kentuckiense* (*H_e_* = 0.522) [38]. The observed genetic diversity was found to be less diverse compared to some species, yet it surpassed that of *C. kanran*, which has a genetic diversity index of *H_e_* = 0.269 [37]. Species with limited distribution ranges typically exhibit lower genetic diversity compared to those with broader distributions. Moreover, endemic, rare, or endangered species are often associated with reduced genetic diversity, attributable to small population sizes, population isolation, and specialization to specific habitats [19]. In addition, this variation of *Cypripedium* species in genetic diversity levels may also be attributed to factors such as environmental conditions, the degree of habitat fragmentation, and other related influences.

Environmental factors such as temperature and precipitation are selective forces, and can affect genetic diversity; however, as genetic variation is mostly studied with anonymous molecular markers, the relative contribution of selective processes to the genetic diversity is difficult to assess [39]. Therefore, the influence of environmental factors on genetic diversity needs to be further studied. In general, habitat fragmentation leads to the loss of genetic diversity, and the longer the history of habitat fragmentation, the greater the loss of genetic diversity [40,41]. In Shangrila, the habitat of *C. flavum* has been fragmented for about 70 years [42], which is not a long period, but with the extension of habitat fragmentation, more genetic diversity would be lost. Inbreeding is one of the negative effects of small populations caused by habitat fragmentation. For orchids, pollen is aggregated into pollinia that usually detach from the anther as a single unit together, and all seeds in an orchid capsule are likely to be derived from a single pollen donor [12], which would easily lead to inbreeding in a small population.

### 3.2. Genetic Structure and Population Differentiation

Orchids, with a minute size of seeds and their capability of long-distance dispersal, have often been reported to have low population differentiation (terrestrial orchid *Cremastra appendiculata F_st_* = 0.066 [43] and *Cymbidium tortisepalum F_st_* = 0.090 [14]; epiphytic orchid *Pseudococcus microcirculus F_st_* = 0.030 − 0.04 [44]). In our study, the estimate of *F_st_* among the populations of terrestrial orchid *C. flavum* was 0.0896, indicating that the populations’ differentiation was weak. The results of STRUCTURE analysis showed that there were 2 genetic groups with no strong genetic differentiation, which is associated with strong gene flow among populations. The STRUCTURE analysis showed that NX and WFS were closer to XRD and XZD, and the clustering of populations did not follow patterns of geographical distribution, which may be due to strong gene flow, lack of geographic isolation, and similar habitat conditions among populations. The results of PCA at the population level showed that there were 2 genetic groups, which was consistent with the results of the STRUCTURE analysis. The results of PCA at the individual level showed that most of the individuals could not be clearly distinguished by population, which was consistent with the low genetic differentiation by AMOVA (*F_st_*= 0.0896). Long-distance, mutual gene flows among populations can link many scattered populations together. The genetic distance between any two populations is not consistent with the spatial distance between populations, indicating that the genetic differentiation between populations does not conform to the model of isolation by distance. Long-distance gene flow among the seven populations or from other populations would lead to no correlation between genetic differentiation and distance. The analysis of Molecular Variance (AMOVA) indicated that the majority of genetic diversity was harbored within populations (91.25%), while a minor proportion was attributable to differences among populations (8.75%). This pattern of genetic variation aligns with patterns observed in outcrossing plant species [14,45]. This suggests that the genetic composition of *C. flavum* is comparatively straightforward, lacking intricate lineage distinctions. The minimal genetic differentiation observed in *C. flavum* could be attributed to its distinctive life history, specialized floral characteristics, an extensive evolutionary past, and the significant bottlenecks it likely endured during the last glaciation. Additionally, the fragmentation of its habitat has likely impacted the dispersal of seeds and pollen [45]. Another reason is that there is a higher gene flow among the populations (*N_m_* = 5.0826). When the value of *N_m_* is less than 1, genetic drift becomes the primary influence on the structure of population genetics. Conversely, when *N_m_* exceeds 1, adequate genetic interchange occurs, mitigating the potential for genetic differentiation that genetic drift might cause [46].

### 3.3. Effects of Habitat Fragmentation

It is generally believed that habitat fragmentation will lead to inbreeding [47]; however, with deceptive orchids, when their pollinators realize they have been cheated, they fly some distance and then pollinate again, which can promote gene flow between populations [48]. In theory, this deceptive pollination mechanism is conducive to the maintenance of genetic diversity. The minute, wind-dispersed seeds of orchids are capable of traversing considerable distances, sometimes over hundreds of kilometers. Orchids tend to exhibit relatively low levels of genetic differentiation, even among widely disjunct populations [12]. For deceptive *Orchis mascula*, among-population gene flow was largely the result of pollen flow, and its pollen flow can reach up to 508 m [13]. Though *C. flavum* is threatened by habitat fragmentation, *C. flavum* still maintains high genetic diversity and gene flow in the present fragmented habitat. Long-distance pollen and seed dispersal is likely to support gene flow even between distant populations, which efficiently delays genetic isolation.

According to our field observation, the fruiting rate of small populations of *C. flavum* is very low, sometimes even zero. Small populations are less attractive to pollinators, and results in inadequate pollination; this reduction in fecundity is referred to as ecological Allee effect [49]. Consequently, *C. flavum* may suffer more from ecological consequences than genetic consequences of habitat fragmentation at present. Many ecological processes caused by habitat fragmentation, such as inadequate pollination and low growth rate of small populations, are the main factors threatening its survival. The protection of this plant should be habitat protection, reducing human disturbance and preventing the further decline of population size.

## 4. Materials and Methods

### 4.1. Plant Material and Study Sites

**Figure 4:**
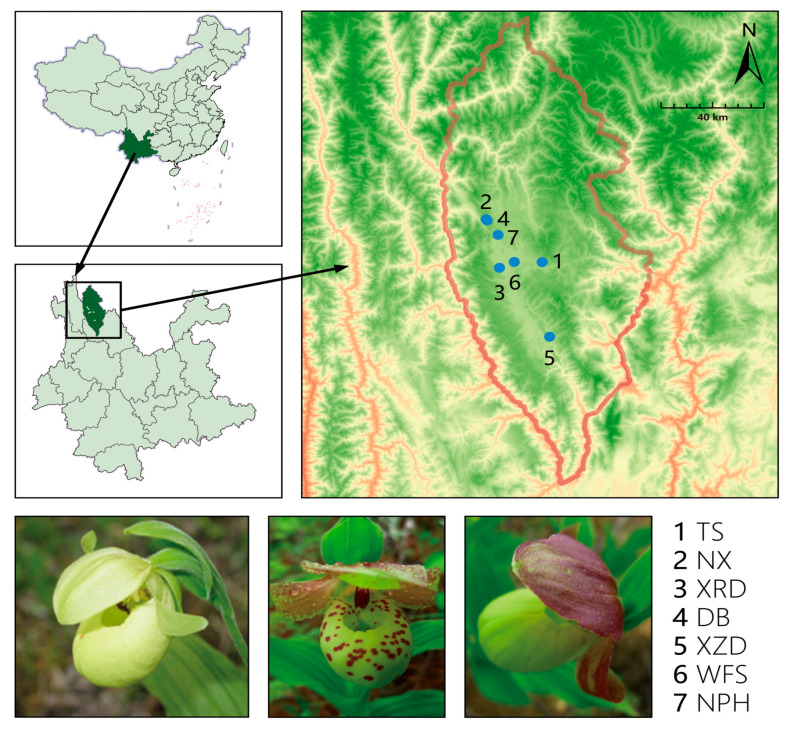
Map of sampling populations and polymorphic flowers of *C. flavum*.

*C. flavum* from different sites were collected (Figure 4) from the northwest of Yunnan Province, China and stored in silica gel. A range of 10 to 28 individuals were sampled in each population, and the sampled individuals were at least 2 m apart to avoid repeated sampling within the same clone. A total of 150 individuals of *C. flavum* were sampled from seven sites in total (Table 5).

### 4.2. DNA Extraction and AFLP Analysis

DNA was extracted from each leaf sample with 50 mg leaf tissues using the NEP003 Genomic DNA Miniprep Kit (Beijing Dingguo Changsheng Biotechnology Corporation, Beijing, China). The quality and quantity of DNA were assessed using the NanoDrop^®^ ND-1000 UV/V spectrophotometer (Thermo Fisher Scientific, Waltham, MA, USA). In TE buffer, containing 10 mM Tris and 1 mM EDTA at pH 8.0, the DNA concentration was meticulously calibrated to achieve a final measurement of 50 ng per microliter.

The DNA restriction–ligation reactions and pre-selective and selective amplifications were performed as described by Vos with some minor modifications [50]. The *Mse* I selective amplification primers were fluorescently labeled with FAM at the 5’ end. Briefly, a 20 μL restriction enzyme digestion and ligation reaction system including MseI (8 U), EcoRI (8 U), T4 DNA ligase (3 U), EcoRI adaptor (30 ng), MseI adaptor (30 ng), and genomic DNA (200 ng) was contained. The digestion and ligation protocol involved operation at 37 °C for 5 h followed by 8 °C for 4 h and 4 °C for more than 8 h. Ligation products from each sample were subsequently diluted 20-fold with TE buffer and used as a template for pre-selective amplification with the primer pair EcoRI + A/MseI +C. PCR reactions were performed in a 25 µL mixture comprising 10 mM Tris-HCl (pH 8.3), 1.5 mM MgCl_2_, 0.2 mM of each dNTP, 25 ng of each primer, 1 U of Taq DNA Polymerase (Boehringer, Ingelheim am Rhein, Germany), and 2 µL of DNA fragments. The PCR conditions were set as follows: an initial denaturation at 94 °C for 2 min, followed by 30 cycles of denaturation at 94 °C for 30 s and annealing at 56 °C for 30 s, 72 °C for 80 s, followed by 72 °C for 5 min, before holding at 4 °C until use. The pre-amplified products were detected by electrophoresis in 1% agarose gel 25 μL. The PCR products of pre-amplification were diluted 20-fold and used as a template for selective amplification with eight EcoRI + 3/MseI + 3 selective primer combinations, which were chosen from sixty-four sets screened using four randomly selected samples. The PCR amplification was performed in a 25 µL volume of 10 mM Tris-HCl pH 8.3, 1.5 mM MgCl2, 0.2 mM of each dNTP, 5 ng EcoRI primer, 30 ng MseI primer, 1 U Taq DNA Polymerase, and 2 µL pre-amplified DNA.

The PCR protocol was established as follows: an initial denaturation step at 94 °C for 30 s, 12 cycles at 94 °C for 30 s, annealing at 65 °C for 30 s with a decrement of 0.7 °C per cycle, and extension at 72 °C for 80 s. This was succeeded by 23 cycles of denaturation at 94 °C for 30 s, annealing at 55 °C for 30 s, and extension at 72 °C for 80 s. A final extension was performed at 72 °C for 5 min, after which the samples were held at 4 °C until further analysis. Capillary electrophoresis of the selectively amplified products was conducted on an ABI 377 genetic analyzer.

### 4.3. Data Analysis

The amplified AFLP fragments were analyzed with the GeneMarker 3.1 analysis software (Soft Genetics, State College, PA, USA). The amplified fragment profiles were assembled in binary format and visually scored for allele presence (1) and absence (0) for all the samples. The threshold for allele calling was set at 100 relative fluorescence (rfu) so that any peaks at 100 (rfu) or higher were assigned as 1, and those lower than 1 were assigned as 0.

The binary matrix, which illustrates the presence or absence of each allele, underwent further analysis utilizing the GenAlEx 6.503 add-in for MS Excel 2016 [51]. This analysis included the observed number of alleles (Na), effective number of alleles (Ne), Shannon’s information index (I), and Nei’s gene diversity (H). The proportion of polymorphic loci, often denoted as PPB, the coefficient of inbreeding within a population (*F_is_*), the total population’s inbreeding coefficient (*F_it_*), the differentiation coefficient among populations known as (*F_st_*), and the measure of gene flow (*N_m_*) are critical genetic parameters. PIC_i_ = 2f_i_ (1-f_i_), where PIC is the polymorphic information content of marker i, f_i_ is the frequency of the fragments that were present, and 1-f_i_ is the frequency of the fragments that were absent [52]. The diversity Müller index (Mu) was determined using the equation Mu = (n * H)/(n − 1), where ‘n’ represents the sample size and ‘H’ denotes Nei’s gene diversity [53]. This index adjusts Nei’s gene diversity for small sample sizes. To assess genetic variation within and among populations, an Analysis of Molecular Variance (AMOVA) was conducted. The significance of the variance components was evaluated with nonparametric permutation tests, employing 1000 permutations for statistical validation [54].

The structure of the populations was assessed using a model-based (Bayesian) analysis in STRUCTURE v2.3.4 [55]. The k-values generated were from four independent analyses of the fixed number of population sub-groups (1–10). The analysis was based on 100,000 Markov chain Monte Carlo (MCMC) iterations and an initial burn-in period of 10,000, with no previous information on the source of accession. The best-fit k-value for the population was calculated using Structure Harvester [56]. In addition, Structure Harvester was used to generate the CLUMPP files for each k-value based on the log probability of the data [LnP(D)] and derived statistics (∆K). The number of sub-populations was determined when the ∆K value reached a maximum [32]. The principal coordinate analysis (PCoA) was performed by GenAlEx 6.503 [51].

## 5. Conclusions

The genetic diversity of *C. flavum* from fragmented habitats in Shangrila was studied. The results showed that genetic diversity within populations was greater than that observed among different populations. Furthermore, minimal genetic differentiation both within and between these populations was detected. The majority of genetic variation in *C. flavum* was found to occur at the intrapopulation level, with only a minor proportion manifesting between populations. This suggests that robust gene flow is likely mitigating the detrimental impacts of habitat fragmentation. These findings enhance our understanding of the population genetic structure of *C. flavum* in its fragmented habitat and will be helpful for developing measures to conserve this vulnerable and endemic species.

## Figures and Tables

**Table 1:** Marker parameters calculated for each AFLP primer pair.

Primer Code	Total Alleles	Total No. of PCR Products	Average Band per Allele	PPB(%)	PIC	Discrimination Power
EA4MC1	74	4770	65.46	100	0.490	19.68
EA4MC4	74	4894	60.42	100	0.493	19.68
EA4MC6	81	5310	68.96	100	0.492	21.54
EA7MC1	77	4968	70.97	100	0.490	20.48
EA8MC5	70	4683	66.90	100	0.494	18.62
Average	75.20	4925	66.54	100	0.492	20.00
Total	376	24652	-	-		-

**Table 2:** Genetic diversity parameters of *C. flavum* populations based on AFLP markers.

Population	Sample Size	*N_a_*	*N_e_*	*I*	*H*	*PPB*	*M_u_*
TS	25	1.926	1.575	0.498	0.334	94.15	0.348
NX	26	1.949	1.613	0.525	0.354	96.01	0.368
XRD	28	1.949	1.596	0.518	0.347	96.28	0.360
DB	17	1.904	1.567	0.494	0.331	92.29	0.352
XZD	22	1.923	1.550	0.486	0.324	94.15	0.339
WFS	22	1.976	1.610	0.529	0.355	98.14	0.372
NPH	10	1.771	1.567	0.489	0.329	88.03	0.366
Total	150	1.914	1.583	0.505	0.339	94.15	0.358

*N_a_*: observed number of alleles; *N_e_*: effective number of alleles; *I*: Shannon’s information index; *H*: Nei’s gene diversity; *PPB*: percentage of polymorphic loci; *M_u_*: Müller index of diversity.

**Table 3:** Genetic differentiation of *C. flavum* populations.

POPGEN					AMOVA					
Sample Size	*F_is_*	*F_it_*	*F_st_*	*N*_m_	Source of Variation	df	SSD	MSD	Variance Components	Total Variance
150	0.3258	0.2966	0.0896	5.0826	Among populations	6	1224.904	203.651	6.443	8.75%
					Within populations	143	9612.236	67.218	67.218	91.25%

*F_is_*: inbreeding coefficient within populations; *F_it_*: overall inbreeding coefficient; *F_st_*: population differentiation; *N*_m_: gene flow.

**Table 4:** Nei’s genetic distance among populations of *C. flavum*.

	TS	NX	XRD	DB	XZD	WFS	NPH
TS	0.000						
NX	0.052	0.000					
XRD	0.106	0.068	0.000				
DB	0.113	0.095	0.042	0.000			
XZD	0.108	0.108	0.075	0.059	0.000		
WFS	0.067	0.060	0.056	0.068	0.056	0.000	
NPH	0.114	0.121	0.112	0.118	0.125	0.076	0.000

**Table 5:** Characters of sampling populations.

Population Code	Altitude(m)	Sample Size	Geographic Coordinates	Habitat Characters
TS	3460	25	27°47′53″ N, 99° 49′ 58″ E	Northeast slope; Shrubbery
NX	3180	26	27°58′ 15″ N, 99°34′ 52″ E	Northwest slope; Popular forest
XRD	3460	28	27° 45′ 57″ N, 99° 39′ 02″ E	Northwest slope; Shrubbery
DB	3170	17	27° 57′ 56″ N, 99° 35′ 11″ E	Northwest slope; Shrubbery
XZD	3360	22	27° 28′ 54″ N, 99° 52′ 51″ E	Northwest slope; Fir forest
WFS	3240	22	27° 47′ 37″ N, 99° 42′ 46″ E	Northwest slope; Shrubbery
NPH	3260	10	27° 54′ 23″ N, 99° 38′ 13″ E	Valley; Sparse forest

## Data Availability

The data presented in this study are available on request from the corresponding author. The data are not publicly available due to ethical restrictions.
